# Molecular Dynamics Computer Simulations of Multidrug RND Efflux Pumps

**DOI:** 10.5936/csbj.201302008

**Published:** 2013-03-03

**Authors:** Paolo Ruggerone, Attilio V. Vargiu, Francesca Collu, Nadine Fischer, Christian Kandt

**Affiliations:** a Department of Physics, University of Cagliari, Cittadella Universitaria S.P. Monserrato-Sestu Km 0.700, 09042 Monserrato (CA), Cagliari, Italy; b CNR-IOM, Unità SLACS, S.P. Monserrato-Sestu Km 0.700, I-09042 Monserrato (CA), Italy; c Departement fu r Chemie und Biochemie, Universita t Bern, Freiestrasse 3, CH-3012 Bern, Switzerland; d Computational Structural Biology, Department of Life Science Informatics B-IT, Life & Medical Sciences Institute, University of Bonn, Dahlmannstr. 2, 53113 Bonn, Germany

**Keywords:** AcrB, AcrA, TolC, MexB, MexA, OprM, antibiotics resistance, membrane protein

## Abstract

Over-expression of multidrug efflux pumps of the Resistance Nodulation Division (RND) protein super family counts among the main causes for microbial resistance against pharmaceuticals. Understanding the molecular basis of this process is one of the major challenges of modern biomedical research, involving a broad range of experimental and computational techniques. Here we review the current state of RND transporter investigation employing molecular dynamics simulations providing conformational samples of transporter components to obtain insights into the functional mechanism underlying efflux pump-mediated antibiotics resistance in *Escherichia coli* and *Pseudomonas aeruginosa*.

## 1. INTRODUCTION

### 1.1. Molecular Dynamics Simulations

While the determination of the three-dimensional structure of a protein is a landmark on the way to understand its function, one key element is still missing, and that is the element of motion. Proteins are in an ongoing state of motion easily exceeding mere thermal fluctuation and in most cases this conformational dynamics is the foundation enabling a protein to carry out its physiological function in the first place [1, 2]. Part of the molecular mechanical branch of modelling techniques [2], molecular dynamics (MD) simulations numerically investigate the motion of a system of particles under the influence of internal (interactions between atoms) and external forces such as temperature or pressure [3] as well as optional additional forces in steered or targeted MD [4]). A key ingredient of MD simulations is the potential energy function that relates energy to structure using harmonic, periodic, Coulomb and Lennard Jones-like potentials to calculate the forces acting on each particle in the system. Employing Newton's second law of motion MD simulation uses this information to predict each particle's motion during the next few femtoseconds. Repeating this step millions of times, a trajectory of all atoms in the system over time is generated [1–3, 5]. Complementing and extending the nearly static experimental 3D data MD simulations bring back for a limited time the element of motion, permitting to cast a glimpse on the dynamics of a (e.g. membrane) protein and its immediate microenvironment at a level of detail not accessible by any experiments today. Moreover, by bringing together a system's components to study their interplay, MD simulations offer a literally synthetic approach of investigation instead of dissecting the system to deduce its functional mechanism.

Since the first MD studies published by Alder and Wainwright more than 50 years ago [6, 7], the first MD simulation of a protein carried out by McCammon and co-workers 20 years later [8], the first simulation of a lipid bilayer by Van der Ploeg and Berendsen in 1983 [9], and the first simulation study of a bilayer-embedded membrane protein by Edholm et al. 17 years ago [10], MD simulations have benefited enormously from the impressive advances made in computer and software development, now permitting the investigation of simulation systems of the size of 10^5^ – 10^6^ atoms on a nanosecond to millisecond time scale [11–13]. Beyond providing high resolution conformational samples of proteins and other biomolecules, MD simulations have also recently been employed as a tool to compare and categorize proteins, adding internal conformational dynamics as a third level of protein classification next to amino acid sequence and protein structure [14].

A key question of any MD simulation is whether the amount of conformational sampling achieved is adequate for the problem under investigation. Whereas for small individual molecules appropriately long simulations can be performed permitting a sufficient sampling of the available degrees of freedom, for large molecules like proteins only a partial sampling of conformational space is possible today [15]. However, partial sampling can already yield valuable insights into protein function providing e.g. a set of configurations near the X-ray structure, based on which conformational sub-populations comprising the entire reaction cycle can be determined [11, 16–19]. Moreover, transportation pathways and interaction sites can be elucidated by analyzing e.g. the dynamics of solvent molecules [20–23]. New mutagenesis candidates can be identified as they undergo for example specific distance changes throughout the reaction cycle [16–19, 24] or impacting protein activity [1, 11, 16, 20–22]. To enhance conformational sampling additional forces can be used biasing the simulation in a steered manner [24–34] or the simulation can be performed running several independent copies of the same system differing only in the random seed numbers used in generating the starting velocities. While stating the respective simulation approaches employed in the studies presented in this review, we refer the reader to the original publications for further-going in-depth information and discussion of the individual methodologies, approximations made and their adequateness for the questions investigated.

### 1.2. RND Efflux Pump-mediated Antibiotics Resistance

The discovery, development and clinical exploitation of antibiotics count among the most significant medical advances in history. However, antibiotics lose their efficiency after a period of months to years [35–37], eventually producing new strains of resistant bacteria, as the continuous application of antibiotics wipes out the cells in a bacteria population sensitive to the drug given. At the same time this effect creates perfect survival conditions for the fraction of bacteria immune to the pharmaceuticals applied. With old antibiotics losing their efficiency faster than new ones can be developed [38], a detailed understanding of the molecular basis of microbial multi-drug resistance is paramount for modern biomedical research. The main mechanisms of action underlying antibiotics resistance include the alteration of the drug, the alteration of the drug target as well the reduction of antibiotics concentration inside the bacterium by lowering influx into and/or enhancing the extrusion out of the organism [39, 40].

**Figure 1:**
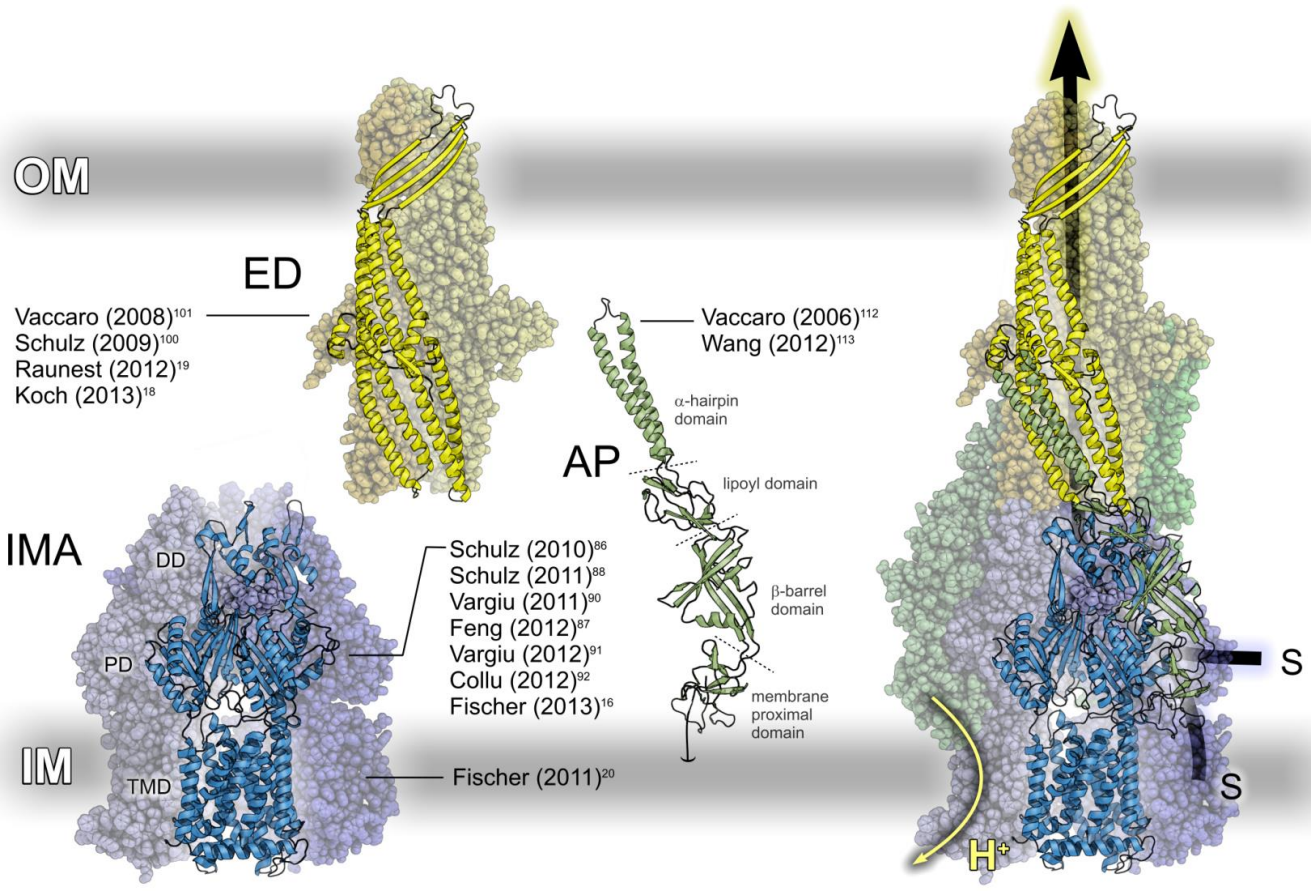
RND efflux pumps comprise three different components (left) assembling into a functional complex (right). Using the proton concentration gradient over the inner membrane (IM) the inner membrane proton / substrate antiporter (IMA) acts as engine and active transporter of the efflux pump, expelling substrates (S) out of the cell via the access-regulated efflux duct (ED) in the outer cell membrane (OM). In the assembled pump IMA and ED are coupled by an inner membrane-anchored adaptor protein (AP), whose actual stochiometry and location in the assembled pump is not known for all RND efflux transporters. To visualize the structure of the assembled IMA-ED-AP complex we used a docking model based on biochemical cross-linking data [41]. The references in the figure represent simulation studies of the respective efflux pump component discussed in this review.

A major way by which Gram-negative bacteria achieve an increased extrusion is through an over-expression of multi-drug efflux pumps of the resistance nodulation division (RND) protein super family [42], preventing drug access to the target molecule [43, 44]. RND transporters function as transiently assembled protein complexes constituting (a) an inner membrane proton / substrate antiporter, functioning as engine and active transporter of the assembled pump (Figure 1, IMA); (b) an access-regulated outer membrane channel acting as efflux duct for substrate trafficking (Figure 1, ED) and (c) an inner membrane-anchored adaptor protein (Figure 1, AP) coupling IMA and ED, enhancing pump activity [45]. Whereas crystal structures have recently become available for all components of three different but structurally homologue RND efflux pumps in *Escherichia* coli (AcrAB-TolC and CusBA-C) [41, 46–57] and *Pseudomonas aeruginosa* (MexAB-OprM) [41, 58–60], the structure of the assembled pump is unknown. The visualization of the assembled IMA-AP-ED complex in Figure 1 shows a docking model based on biochemical cross-linking data [41]. Whereas this model comprises three APs interacting with IMA and ED, recent studies suggest that MexA and AcrA form a funnel-like hexamer when binding to their respective EDs [61–63] similar to the IMA-AP crystal structure of the heavy metal efflux transporter CusBA [57].

## 2. EFFLUX PUMP SIMULATIONS

With the advent of high-resolution crystal structures, computer simulations have grown into a vivid field of research in investigating the functional mechanisms of efflux pump-mediated antibiotics resistance, employing a palette of computational methodologies including elastic network normal mode analyses [59, 64], multiple basin [65] and MD simulations. In this review we focus on computational studies of RND efflux transporter components using molecular dynamics simulations. Reflecting the general structure of an RND efflux pump (Figure 1), we organized the review part in three sections, summarizing the simulation studies reported for the inner membrane antiporter (section 2.1), the outer membrane efflux duct (section 2.2) and the adaptor protein (section 2.3).

### 2.1 Inner Membrane Antiporter

Engine and active transporter of the assembled efflux pump, the inner membrane proton / drug antiporter is a homo-trimer whose individual protomers are organized in three distinctive sections, each fulfilling different functions (Figure 1, IMA). Whereas energy conversion via proton conduction takes place in the trans-membrane domain (TMD), substrate recruitment and transport mainly occur in the periplasmic porter domain (PD) which in turn is coupled through the docking domain (DD) to the outer membrane ED (Figure 1), or to the hexameric assembly of APs in the constituted pump. A characteristic IMA feature is a structural asymmetry among the monomers, each trapped in a different conformation, interpreted as reaction cycle intermediates “**L**oose” / “access” (monomer A), “**T**ight” / “binding” (monomer B) and “**O**pen” / “extrusion” (monomer C) in a peristaltic pump functional mechanism [55, 56, 66]. IMA simulation studies published so far have focused on two questions: How are protons transported (section 2.1.1)? How is substrate transported (section 2.1.2)? As at the time of writing MD studies of the heavy metal efflux transporter CusA have not been reported yet, this section focuses on investigations carried out for AcrB and MexB.

#### 2.1.1 Proton transport

As proton conduction in proteins occurs along hydrogen-bonded networks of polar residues and water molecules [67] in a Grotthuss-like mechanism [68–71], knowledge of the protein-internal water distribution and interacting residues allows drawing conclusions to possible pathways of proton conduction [21, 22, 72–76].

**Figure 2:**
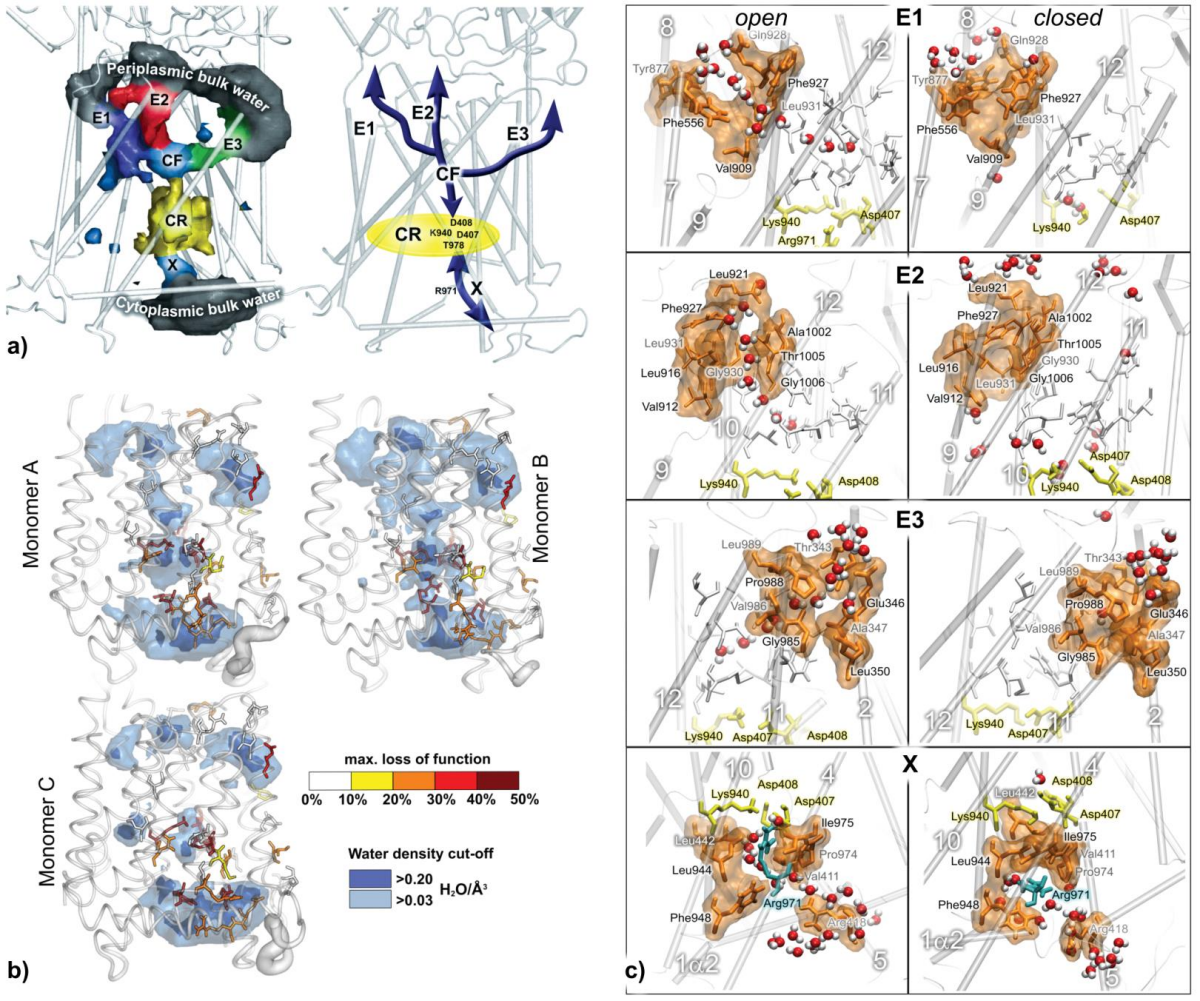
On a 50 ns time scale WT AcrB displays a TMD-internal water distribution suggesting three alternative routes of proton transfer where the key residue-comprising core region (CR) is connected to bulk water via one cytoplasmic (X) and three periplasmic water channels (E1-3) merging in single conflux region (CF) (a). Dynamic and monomer-specific TMD hydration was found in agreement with the location and impact of known point mutations (b) with bulk water access regulated by four groups of gating residues (c). Adapted from [20], modified.

In AcrB the protein-internal water distribution is experimentally unknown and so far five TMD residues have been identified whose mutation to alanine leads to a function loss of 90% or more [52, 77–80]. Furthermore, for each monomer an intermediate-specific protonation scenario has been proposed based on the available X-ray structures [81]. To predict TMD hydration and potential new key residue candidates Fischer and Kandt performed a series of 6 x 50 ns independent and unbiased atomistic MD simulations of asymmetric, wild-type (WT) AcrB in a phospholipid / water environment, simulating each monomer in its currently proposed protonation scenario [20]. Using the MD trajectories to compute spatial residence probabilities of TMD-internal water, the authors find that TMD water is organized in one cytoplasmic and up to three periplasmic water channels connecting the known five key residues to bulk phase, suggesting three alternative routes of proton transfer (Figure 2a). Reflecting the different protonation scenarios in each monomer, the TMD water distribution is reported to be intermediate-specific and correlating well with the location of 15 experimentally tested residues [52, 77–80] and their respective impact on AcrB function (Figure 2b). Using different time resolutions in computing the water densities, the authors find the water channels dynamic and their bulk water access regulated by four groups of gating residues in a combination of side chain re-orientations preceded by intermediate-specific shifts of α-helices enabling or disabling opening or closure of the gating residues (Figure 2c).

#### 2.1.2 Substrate transport

Computational studies assessing the question of substrate transport in the proton/drug antiporter focus on the dynamics of the porter domain, using unbiased or steered MD in the absence (section 2.1.2.1) or presence of substrate (section 2.1.2.2).

##### 2.1.2.1 IMA dynamics in the absence of substrate

**Figure 3:**
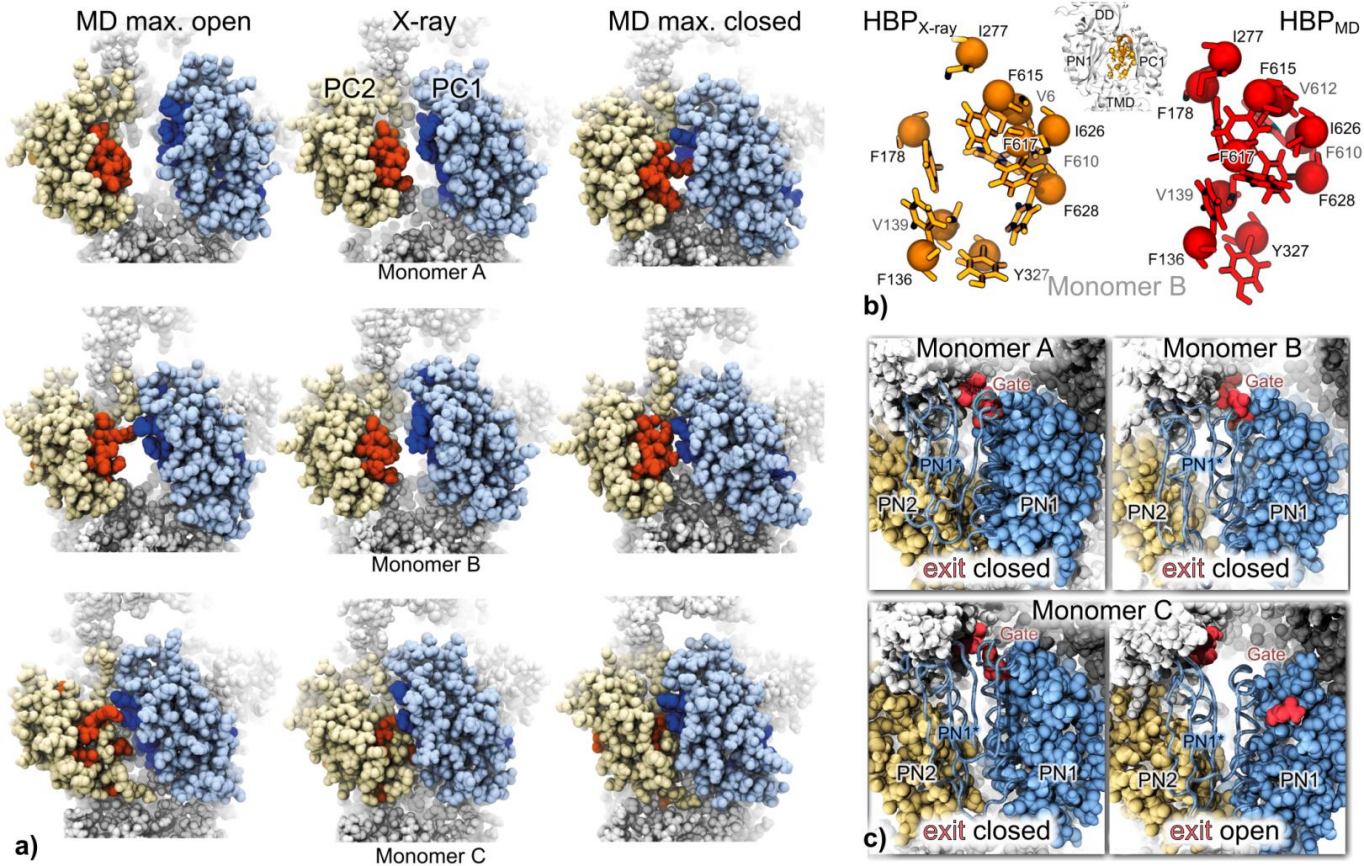
On a 200 ns time scale WT AcrB's drug-transporting porter domain is highly flexible, displaying opening and closing motions of the proximal binding pocket in monomers A and B (a), a closure of the further inward located hydrophobic binding pocket (HBP) in B (b) and an opening and closing of the proposed exit of the drug transport channel in C (c). Adapted from [16], modified.

Focusing on PD ground state dynamics in the absence of substrate, Fischer and Kandt [16] carried out a series of 6 x 100 ns independent and unbiased atomistic MD simulations of asymmetric WT AcrB in a phospholipid membrane / water environment to address the question why all 34 currently available AcrB crystal structures [47, 51–53, 55, 56, 82] exhibit very similar PD conformations. Displaying Cα RMSDs below 1 Å after superposition to the simulation starting structure [55], in all crystal structures the outer *access* or *proximal binding pocket* PBP [47, 53] is open in monomers A and B but closed in C, while the inner *deep, distal or hydrophobic binding pocket* HBP [47, 53] is open in B but closed in A and C. At the same time the exit region of the PD substrate transport channel (PDx) is closed in monomers A and B but open in monomer C (Figure 3). Observing opening and closing motions of the PBP in monomers A and B (Figure 3a), a predominantly closed HBP in all monomers (Figure 3b) as well as an opening and closing PDx in monomer C (Figure 3c), Fischer and Kandt proposed that the X-ray conformations are stabilized by a component absent in the simulations, suggesting bound but unresolved substrate molecules as possible explanation. Based on the observed conformational dynamics the study further suggests that each of the known three reaction cycle intermediates occurs in at least two variants and the Thr676 loop independently regulates porter domain access likely playing a key role in substrate transport. If the proximal binding pocket dynamics have an inhibiting effect on AcrB pump activity by lowering the life time of substrate-accessible conformations, the observed dynamics could provide a structural explanation for the AcrB activity-enhancing effect of the adaptor protein AcrA [45] stabilizing PC1 and PC2 subdomain orientations.

##### 2.1.2.2 IMA dynamics in presence of substrate

The picture extracted from the crystallographic data is an invaluable starting point to understand substrate-IMA interactions. However, a complete picture must include the dynamics of all the parts involved, i.e., transporter, substrate, and solvent. Unfortunately, experiments aimed at estimating the efflux kinetics are quite complex and possible so far only for β-lactams antibiotics [83, 84]. In addition, despite the strong effect of efflux on the minimum inhibitory concentrations (MICs) of substrates, it is very difficult to quantitatively determine the contribution of drug transport among all factors affecting the susceptibility of a cell to antibiotics [83, 84]. Computer simulations are thus an important tool to complement and interpret experiments on kinetics [85]. A first question concerns the plausibility of the functional rotation. By mimicking the conformational transitions of the AcrB reaction cycle via targeted molecular dynamics (tMD) [31], Schulz et al. [86] observed in 4 independent simulations a displacement of doxorubicin by 8 Å from the HBP towards PDx. Concurrently, a zipper-like closure of the HBP was observed, supporting the peristaltic pump mechanism proposed on the basis of the crystal structures [47, 51–53, 55, 56, 82]. Insights into the behaviour of the solvent during the imposed functional rotation were achieved in additional unbiased [87] and tMD simulations [88], detecting a directed water flow towards the PDx. This direction is defined by the conformational changes of PD. However, Schulz et al. never observed a complete extrusion of doxorubicin. One possibility to explain this is that the passage of the drug through PDx might be, at least partially, diffusion-driven, and thus should occur on a time scale much larger than that captured by all-atom MD simulations. In addition, the motion of the drug might further be enhanced by the presence of other substrates. Finally, how other proteins components absent in the simulations affect transport needs to be understood better, in the long run leading to eventually taking into account the entire efflux pump. Similar results were obtained by Feng and co-workers [87] who investigated the in silico dynamics of AcrB in complex with erythromycin, rifampicin and minocycline. The authors found that rifampicin and erythromycin, bound to the A monomer, made a unidirectional peristaltic movement towards the extrusion funnel of ToIC, which was facilitated by water flux within the channel of AcrB. Minocycline in the B monomer moved from the distal binding pocket towards the gate of the central funnel.

A key point to the comprehension of efflux systems regards the link between affinity and efficient extrusion: how high should a compound's affinity to the transporter be to make it a good substrate? A substrate should remain inside IMA long enough to be extruded but its affinity should not be too high, otherwise the extrusion might be overly energy-demanding. Site-directed mutagenesis studies provided the experimental basis to shed some light on this issue. Bohnert et al. [89] systematically mutated HBP phenylalanine residues into alanine and determined the mutants’ susceptibility to various antimicrobials. Interestingly, the F610A point mutation displayed the most significant impact on the substrates’ MICs, while replacing other HBP phenylalanines with alanines had smaller and more variable effects. Puzzling in these results is that F610 does not directly interact with doxorubicin and minocycline in the crystal structure [51] and it is practically not involved in the zipper-like movement of the HBP residues responsible of the departure of doxorubicin from the pocket as described above [86]; and doxorubicin displays one of the most pronounced MIC reductions in the F610A mutant [89]. Combining several computational techniques, Vargiu and co-workers [90] provided a possible explanation for the role of F610, as in the mutant the authors found doxorubicin sliding deeply into the binding pocket, thus increasing the strength of the protein-compound interaction and making extrusion hardly feasible. Indeed, during subsequent tMD simulations of the AcrB reaction cycle, in the mutant doxorubicin was either not extruded from the binding site or displaced along a direction other than the one associated with extrusion. In WT AcrB F610 provides the appropriate balance between affinity and energy requirement to extrude a substrate. The study indicates how subtle interactions determine the functionality of multidrug transporters, since decreased transport might not be simplistically correlated to decreased substrate binding affinity [90].

Using a truncated protein model restricted to the porter and docking domain, Vargiu and Nikaido simulated AcrB in complex with substrates, non-substrates, and inhibitors previously docked to the HBP [91]. While all substrates tested remained bound to the HBP, the authors found that non-substrates, predicted by the docking procedure to bind outside the HBP, remained there during 50 - 80 ns of unbiased MD. Moreover, the two AcrB inhibitors (Phe-Arg-β-naphthylamide and 1-(1-naphtylmethyl)-piperazine), located by docking runs inside the HBP, tended to leave the pocket at least partially, straddling the G-rich loop whose flexibility has been indicated by Yamaguchi and co-workers to be essential for the functioning of AcrB [53]. Importantly, MD simulations by Feng et al. [87] confirmed that the mutations of G616P and G619P could prevent the movement of the G-loop.

Whereas at the time of writing 34 crystal structures have been reported for AcrB [47, 51–53, 55, 56, 82], only one X-ray structure of the apo protein has been published for its *P. Aeruginosa* homologue MexB [60]. With a sequence identity of 69.8% MexB and AcrB are structurally very similar, sharing several conformational key features. However, in monomer A the proteins differ in their respective PBP conformation, which is open in AcrB but closed in MexB, hindering substrates to enter. It is currently unknown whether the different PBP conformation in AcrB and MexB is an effect of the crystallization procedure, an indication of a different monomer involvement in the extrusion process, or an evidence supporting the high PBP flexibility proposed in [16].

**Figure 4:**
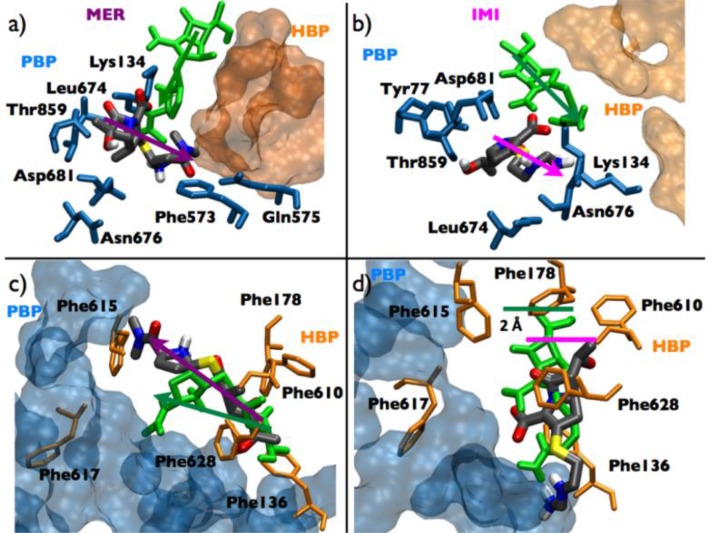
Configurations assumed by MER and IMI in MexB according to 50ns-long MD simulations. The residues of MexB are in licorice colored according to the region they belong to (blue, PBP; orange, HBP). Panels a and b refer to the compounds in PBP, c and d in HBP. The starting configurations of the carbapenems are represented in green licorice, those at the end of the simulations in atom-code colored licorice. Arrows denote the orientation of the compounds in panels a, b, and c. In panel d we report the shift of IMI. Adapted from [92], modified.

Imipenem (IMI) and meropenem (MER) of the carbapenem compound family have been the most active broad-spectrum antibiotics against *P. aeruginosa* infections [93], but resistant strains have appeared [93–95]. Several studies evaluating compounds’ MICs indicated that MexAB-OprM affects the activity of MER, while that of IMI is essentially insensitive to over-expression of the pump [96–99]. To identify the molecular basis of the underlying carbapenem–efflux-pump interactions Collu and co-workers performed docking and 8 standard 50ns-long MD simulations using a truncated model of MexB [92]. Configurations assumed by the compounds during the simulations are reported in Figure 4. Whereas MER showed high affinity to the HBP, assuming there conformations that prelude to efficient transduction towards the extrusion channel (Figure 4c), IMI did not bind to the HBP with good affinity, exploring geometries similar to those reported in AcrB mutants for poorly transducing substrates (Figure 4d) [90]. The authors suggest two main reasons for these behaviours. First, the bulky and more hydrophobic groups in MER favour interactions with the aromatic-hydrophobic environment of HBP, whereas the more flexible and more hydrophilic tail of IMI does not. Secondly, the interaction with the solvent plays a role. Despite the compounds are highly solvated in both PBP and HBP, the water dynamics around MER is significantly different in HBP than in the bulk solvent. On the contrary, IMI shows the same interactions with solvent inside the HBP and in the bulk.

### 2.2 Outer Membrane Channel

Once recruited by the IMA, the substrate is transported out of the cell via the ED in the assembled pump (Figure 1). Essentially resembling the shape of a hollow cylinder, the ED occurs in at least two different states, blocking the passage of substrate, e.g. when not interacting with an IMA, and permitting the trafficking of substrate for example as part of the assembled RND efflux pump. The underlying gating mechanism has been the main focus of ED simulation studies, which at the time of writing have been reported for *E. coli* TolC and *P. aeruginosa* OprM.

**Figure 5:**
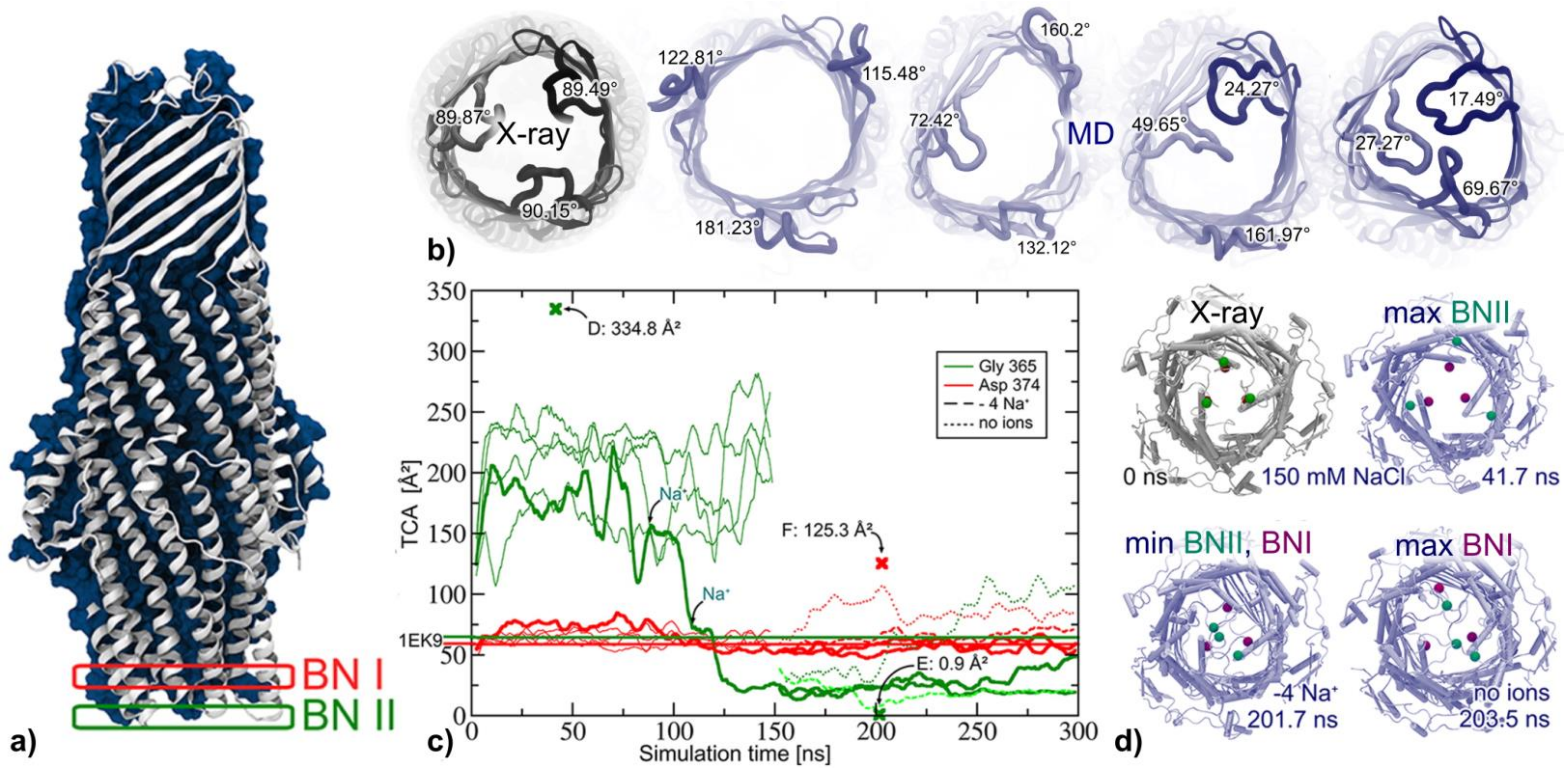
On a 300 ns time scale WT TolC (a) opens and closes freely on extracellular side (b) and in the region of the outer periplasmic bottleneck BNII at Glu365 while the inner bottleneck BNI remains closed unless all NaCl is removed from the system (c, d). Adapted from [19], modified.

Computational studies of TolC focused on MD simulations comparing wild type (WT) and mutants in the outer periplasmic bottleneck region [100, 101], WT ground state dynamics [19] as well as elastic network normal mode analyses exploring possible opening mechanisms using TolC and OprM crystal structures [102]. In a 20 ns MD study of WT and Y362F + R367S TolC Vaccaro and co-workers reported the mutant exhibiting heightened flexibility in the periplasmic mouth region while for the extracellular loops a gating function was proposed based on the observed closing motions [101]. In a series of 20 – 30 ns MD simulations of WT, Y362PF + R367E and Y362F + R367D TolC Schulz and Kleinekathöfer observed WT-like closed periplasmic mouth conformations stabilized by potassium ions coordinated by T152, D153, and E/D367 in the mutant structures [100]. Only when the potassium binding sites were emptied using an outer electric field a BNII (green rectangle in Figure 5a) opening trend was observed.

Simulating WT TolC (Figure 5a) in a series of 9 150 - 300 ns unbiased and independent atomistic MD runs, Raunest and Kandt [19] observed free opening and closing motions on extracellular side (Figure 5b), opening and sodium-induced closing motions of the outer periplasmic bottleneck region [103] (Figure 5c, d green) whereas the inner periplasmic bottleneck [104] remained in a crystal structure-like closed conformation unless all NaCl was removed from the system (Figure 5c, d, red). In that case a re-opening of the outer bottleneck occurred, concurrent with a beginning opening trend of the inner bottleneck. The free opening and closing of the extracellular loops suggested the absence of a gating mechanism on this side as well as hinted at the possibility of designing a novel group of TolC-directed drugs specifically targeting the protein interior. Additionally, the observed conformational dynamics on the opposite side indicates that TolC is locked only on periplasmic side in a sodium-dependent manner. In a similar study Koch et al. [18] sampled the ground state dynamics of WT OprM in a series of 5 independent, unbiased 200ns atomistic MD runs. Like TolC, the OprM simulations suggested unilateral access regulation, with the protein opening and closing freely towards the extracellular while on periplasmic side only the Asp416 region is involved in channel gating. Contrary to TolC, no evidence was found suggesting a Na-dependent lock mechanism in OprM, although for OprM too new sodium binding sites were reported.

### 2.3. Adaptor Protein

Though the third component of the efflux systems, the adaptor protein (Figure 1, AP) (AcrA for *E.coli*, MexA for *P.aeruginosa*), has also been the object of thorough experimental study, several aspects remain unclear, especially regarding the interplay among and the assembling of the three efflux pump components [50, 105–111]. Anchored to the inner membrane, APs extend into the periplasm acting as a central linker between IMA and ED and play a critical role in the transport event itself. However, the apparently simple question of how many AP proteins are necessary in the assembled functional pump has not received a clear-cut answer. Recent studies suggest that both MexA and AcrA show propensity to form a funnel-like hexamer when APs bind to the respective EDs [61, 62], coinciding with the stochiometry seen in the crystal structure of the structurally homologue heavy metal efflux transporter CusA solved in complex with its AP CusB [57].

At the time of writing two AP MD studies have been published: one on MexA [112], the other on AcrA [113]. Vaccaro et al. [112] investigated MexA in the absence of the membrane anchor and without a large part of the membrane proximal (MP) domain, at that time not resolved. Principal components analysis of the 25ns-long MD trajectories identified a hinge-bending motion and a rotation of the α-helical hairpin relative to the other domains of MexA as the two dominant motions. According to the root mean square fluctuation of each residue from its time averaged position the largest fluctuations are for the loop between the two α-helices forming the hairpin, and for two loops in the β-barrel domain. Interestingly, the first two loops (i.e. the hairpin and one of the two β-domain loops) appear to be correlated in their motion. Further, the motion of the helical-hairpin loop appears to correlate with the C-terminal region. Of interest, this region has been shown experimentally to be involved in AP / IMA interactions [114]. These motions indicate considerable flexibility, which is likely to be exploited in the adaptor function of MexA during the assembly and opening of functional pores during pump activity. The simulations offered first interesting insights into the dynamical role of AP, although the study was limited by short simulation times, an incomplete AP structure, no membrane environment and by the fact that only a single MexA protein was considered. Note that the importance of the MP domain has been demonstrated by the recently solved crystal structure of MexA, in which the MP domain adopts two distinct orientations with respect to the other part of the protein [41].

Performing 20 ns MD simulations of WT and mutant AcrA in an aqueous environment under different pH conditions, including the homology-modelled MP but lacking the membrane-anchoring N-Terminus, Wang and co-workers [113] showed that AcrA flexibility largely stems from the α-hairpin and MP domains, whereas the lipoyl and β-barrel domains form a relatively rigid module. The authors further reported that both point mutations and pH influence protein dynamics, with pH 5 conditions reducing conformational flexibility, in agreement with electron paramagnetic resonance experiments [115]. Situated in the ß-barrel domain H285 was identified as regulatory key of the pH-induced changes in conformational flexibility whose reduction could be interpreted as favouring intermolecular packing and reducing the entropy cost of oligomerization. Furthermore, as AcrA/B binding affinity is pH-dependent [61], periplasmic pH changes accompanying the drug efflux could also act as a signal regulating the assembly of the functional AcrAB-TolC complex.

## 3. CONCLUDING REMARKS

In this review we provide a survey on the application of atomistic simulations to study the molecular bases of RND efflux pump-based antibiotics resistance, summarizing the recent studies investigating the conformational dynamics of the inner membrane proton/drug antiporters AcrB and MexB, the outer membrane efflux ducts TolC and OprM as well as the inner membrane-anchored adaptor proteins AcrA and MexA. With the first relevant simulation study published merely seven years ago, the computational investigation of efflux pump-meditated multidrug resistance is still a young field of research that has only just begun to gain momentum. Nonetheless, some interesting findings have already been reported and it will be exciting to see what the future holds for this branch of computational research, already addressing biological questions on a time and system complexity scale that would have been considered impossible only a few years ago.
